# Pre-orthodontic restorative treatment of microdontia diastema teeth using composite injection technique with a digital workflow–Case report

**DOI:** 10.1016/j.heliyon.2023.e15843

**Published:** 2023-04-28

**Authors:** Romain Milian, Etienne Lefrançois, Anastasia Radzikowski, Samuel Morice, Marie Desclos-Theveniau

**Affiliations:** a Private Practice, Cesson-Sévigné and University Hospital, Rennes, France; b Department of Dentistry, University Hospital, Rennes, France; c Department of Orthodontics, University Hospital, Rennes, France; d Registered Dental Technician, Private Dental Laboratory ARGOAT, Ploumagoar, France; e U1317 INSERM, INRAE, Univ Rennes 1, CHU de Rennes, Nutrition Metabolisms and Cancer, Department of Dentistry, University Hospital, Rennes, France

**Keywords:** Composite, Digital workflow, Injection technique, Microdontia, Restorative treatment

## Abstract

Restorative treatment of microdontia teeth is often considered as the final step of post-orthodontic treatment. Based on digital workflow, this clinical report presents pre-orthodontic reshaping of anterior teeth in the smile disharmony of a young patient using bilayering composite injection technique. Transparent silicone indexes for dentin and enamel fillings were fabricated from three-dimensional-printed models of the digital wax-up. This noninvasive, simple and straightforward injection technique was able to provide semipermanent reversible aesthetic restorations while awaiting for adulthood and definitive prosthodontic solution. Closure of diastemas before orthodontic treatment were carried out to restore functional contact point and to guide future teeth movements.

## 1 Introduction

Microdontia is used to describe teeth with altered shape and reduced size [1]. Lateral incisor and third molar are the most common teeth affected by this anomaly. Impaired teeth have a conoid shape with a reduced mesiodistal width which create diastema leading to aesthetic smile disharmony. Such anomalies can create discomfort or hamper patient psychology. Multidisciplinary approach including orthodontic and restorative dentistry are often required to both redistribute space and reshape teeth [2,3]. Defined as PGO (Prosthetically Guided Orthodontics Concept) concept, this digital approach allows teeth restorations before starting orthodontic treatment in order to guide teeth movement [4,5].

There are several treatment options to correct microdontia. Direct restoration with composite resins, composite or ceramic veneers, or full coverage crown can be used to close diastema. Considered as minimally invasive dentistry, resins allow reversible treatment with excellent aesthetic outcome, good clinical longevity and low cost. In adolescents where growth is not fully completed, composite restorations are chosen as first intention to avoid tooth movement [2,6]. It has been shown that resin survival rate was 93% after 4 years when they were performed after orthodontic treatment [7]. Among several restorative technique, stratification consists of superimposing layering composites to mimic the natural anatomy and appearance of a toothshade [8]. To obtain an aesthetic outcome with shade matching, clinician has to select enamel and dentin conventional composite materials by focusing on color properties such as lightness, chroma, hue and translucency [9]. These direct free-hand composite restorations also require a clinician ability and a long chair-time for the patient. With the improvement of flowable composites highly filled, the composite injection technique has emerged to facilitate the application of resin material and to avoid a practitioner-dependent outcome [[10], [11], [12], [13]]. Its protocol has been described recently in several articles [2,[14], [15], [16], [17], [18], [19], [20], [21], [22]].

Contrary to previous publications, this case report aimed to present this technique before orthodontic treatment with consensus between the orthodontist and the prosthodontist to recover an aesthetic disharmony caused by microdontia. A complete digital workflow combining orthodontic and restorative planning were performed and stratifications using bilayering composite injection technique was used to optimize aesthetic outcome [18].

## 2 Clinical report

**Fig. 1:**
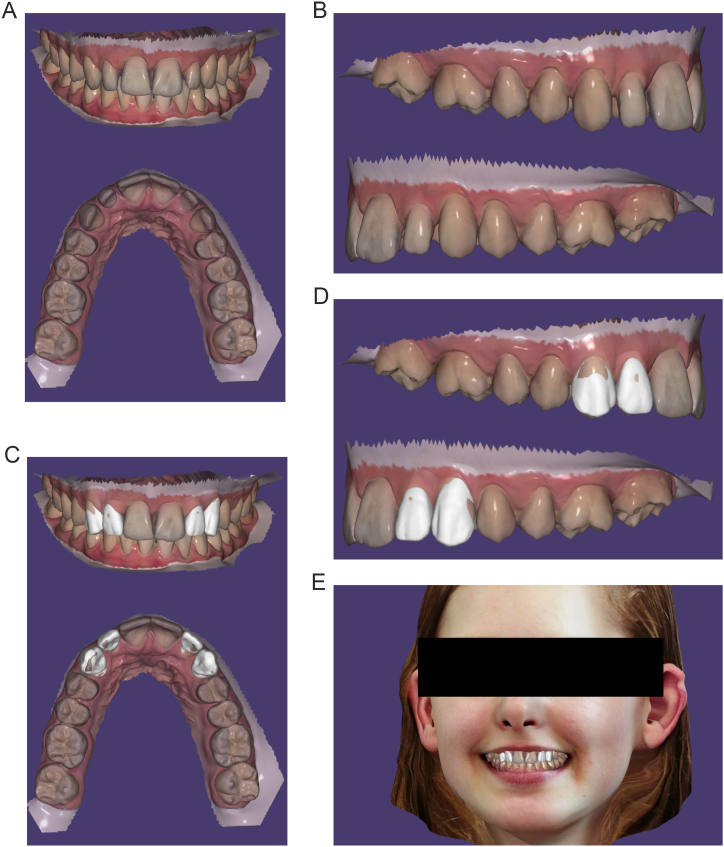
Overview of digital orthodontic setup. A, and B, without virtual digital wax-up. C, D, and E, with virtual digital wax-up (E, Virtual digital wax-up created in 3D virtual patient).

A 16-year-old female patient was referred to Department of Dentistry at the University Hospital of Rennes for dento-maxillary disharmony. Her medical history indicated good general health. Intraoral and extraoral photographs and digital impressions (Trios 3, 3Shape A/S, Copenhagen, Denmark) were taken to elaborate therapeutic approaches. Using OrthoAnalyzer (3Shape, Copenhagen, Denmark), a virtual orthodontic setup was performed by displacing each individual tooth to create harmonious arches aligned with an aesthetic and functional occlusion [23]. Clinical examination and digital setup revealed diastemas on the maxillary lateral incisors and canines due to microdontia (Fig. 1A and B). Virtual setup showed that the mesiodistal widths for both 12–13 and 22–23 would still unmodified at the end of the orthodontic treatment. To facilitate management and teeth movement, the orthodontist and the prosthodontist decided to restore the anatomy and the shape of 12-13-22 and 23 with no-preparation direct restorations using injected composite technique before commencing the orthodontic treatment. The patient gave written informed consent for therapeutic procedure and for the publication of this report. Approval was obtained from the Ethical Committee of the University Hospital (CHU) of Rennes.

**Fig. 2:**
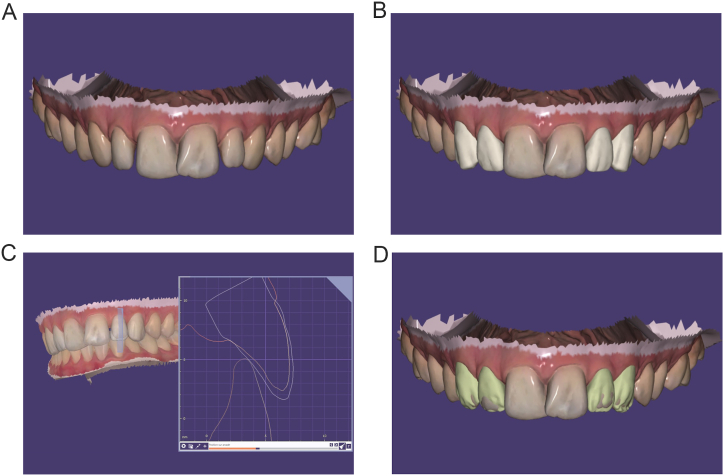
Overview of digital wax-up before orthodontic treatment. A, Initial situation. B, Digital wax-up with dentin core and enamel layer. C, Longitudinal cut of maxillary left lateral incisor to evaluate thickness of restoration. D, Digital wax-up with dentin core.

Digital data from initial arches, orthodontic setup and facial scanning (Bellus3D® extraoral scanner) were imported, superimposed and merged by the exocad DentalCAD software (exocad GmbH) to create a diagnostic wax-up and a virtual smile design (Fig. 1C–E). The wax-up were first performed on digital orthodontic setup to check that the composite restorations would not alter the orthodontic treatment, occlusion and final result. Anatomy of teeth were automatically recreated by the software and then manually shaped in order to obtain an aesthetic outcome (Fig. 1C–E). Final volume restorations were then transferred on the initial digital arch (Fig. 2A and B). Virtual wax-up revealed that the occlusal thickness of both lateral incisors had to be increased at most by 1 mm (Fig. 2C). To optimize the aesthetic appearance, it has been decided to use a layering technique on each tooth by superimposing two layers of composite resins: dentin and enamel. The dentin layer, responsible of the hue and chroma of the tooth allowed the restoration of the dentin core. The enamel layer restored dental anatomy and aesthetic by playing on the superposition of the shades and on the optical properties of the chosen composites. In young teeth, enamel is thick, opaque with high value and low translucency. The dentin core was designed by removing digitally the enamel layer (Fig. 2D).

**Fig. 3:**
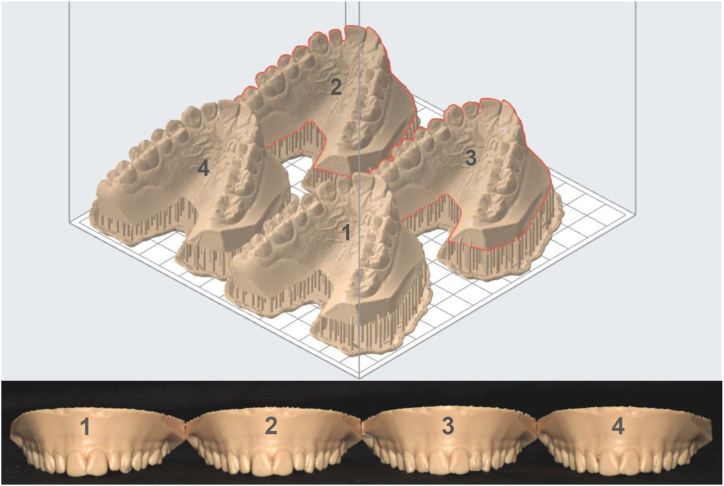
Digital view of 3D printed models and 3D printed models. Canines with dentin core (n°1) and with enamel layer (n°2). Incisors with dentin core (n°3) and with enamel layer (n°4).

It has been decided that canines would be the first teeth to be restored with injected composite. Two models based on canine digital wax-up were 3D printed (Formlabs Form 3B printer) with high precision (0.025 mm): the first for dentin filling and the second for enamel filling (Fig. 3). For both, the natural anatomy of the lateral incisors was preserved in order to correctly place silicone indexes. The same protocol was then followed for the lateral incisor restorations, but including wax-up restored canines (Fig. 3). From 3D printed models, four transparent silicone indexes were made using a clear polyvinylsiloxane (Exaclear, GC Corp., Tokyo, Japan) [17]. The step light-curing of transparent silicone indexes should have been carried out under high pressure to avoid formation of bubbles on the index inner surface [17]. However, it was verified that no bubbles interfered with the volume and surface restorations.

**Fig. 4:**
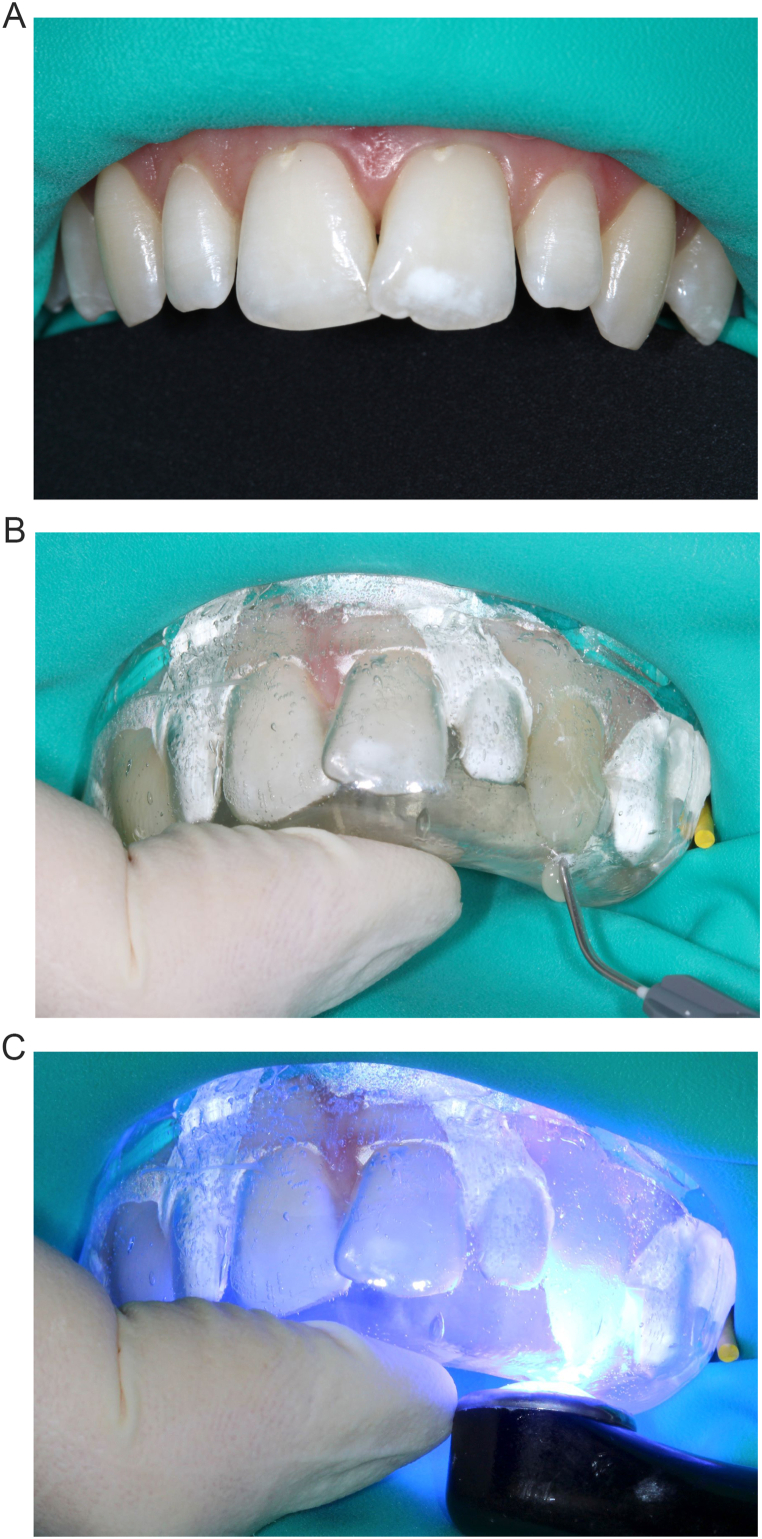
Composite injection technique on canines. A, Rubber dam placement. B, Composite injection through incisal hole of the silicone index. C, Light-curing though the silicone index.

Before the dehydration of the tooth, the enamel and dentine shade of the teeth were determined with a composite button try technique. The choice was A2 (G-ænial Universal Injectable, GC Europe) for dentin-shade and A1 for enamel-shade (G-ænial Universal Injectable, GC Europe). Operative field was isolated with rubber dam after silicone index fitting (Fig. 4A). Four successive injection steps were performed: 2 for canines followed by 2 for lateral incisors. For each of them, the operative sequence was identical [2,[16], [17], [18]]. Briefly, adjacent teeth were covered with Teflon. Restored teeth were etched with 37% orthophosphoric acid (Ultra etch, Ultradent, USA) for 30 seconds, rinsed for 30 seconds with water and air-dried. Universal adhesive (G-Premio Bond, GC Europe) was applied for 20 seconds, air-dried and light-cured for 20 seconds. The dentin silicone index was placed and a dentin-shade (A2) flowable composite (G-ænial Universal Injectable, GC Europe) was injected (Fig. 4B). Resins were light-cured for 30 seconds from both labial and palatal direction (Fig. 4C). After removing the silicone index, excess material was cut off with scalpel. The enamel silicone index was then placed. An enamel-shade (A1) flowable composite (G-ænial Universal Injectable, GC Europe) was injected and light-cured for 30 seconds. Silicone index was removed and any excess composite were cut off with a scalpel. Restorations were light-cured for an additional 60 seconds from each side. A first finishing was made with diamond finishing burs.

**Fig. 5:**
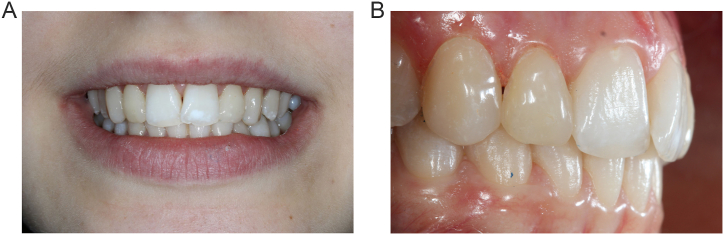
Immediate post-operative views. A, Final photograph of patient's smile. B, Right side view in occlusion.

The same technique was then done for lateral incisor restorations using their specific indexes. After finishing all composite injections, the operative field was removed. Teeth surface and interproximal sites were polished with diamond finishing burs and interproximal sandpaper strips, respectively. A minimal trimming and occlusal adjustments were made. A last polishing of teeth was performed with coarse-gritted finishing discs (Sof-Lex Pop On XT, 3 M Espe) and a two-step diamond polishing system (Kompoline Spirale, Komet France) including a first step with a fine finishing wheel for 30 seconds and then a final lustrous surface with a super fine high-gloss polishing wheel for 30 seconds. The immediate result presented in Fig. 5 is consistent with the digital wax-up. Due to the dehydration of the teeth caused by the rubber dam isolation, the final outcome showed a whiter appearance of the teeth compared to restorations. Dehydration of teeth reduced the teeth's translucency and increased their luminosity.

**Fig. 6:**
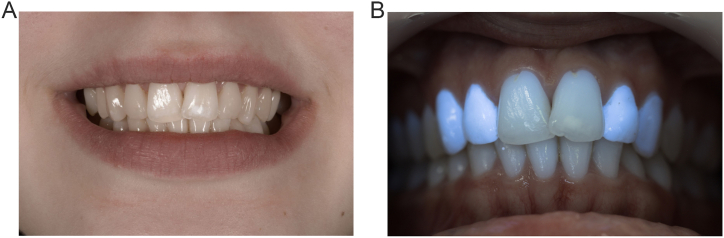
Views after 8 months. A. Patient's smile after re-polishing. B, Fluorescence of restorations in the upper anterior teeth under UV light.

A maintenance appointment was realized after 8 months. The aesthetic outcome was stable with no signs of restoration wear or soft tissue inflammation. To eliminate some staining throughout the restoration surfaces and mostly to increase the survival of the restorations, finishing and polishing procedures were made following the same steps as previously described. The final photograph taken revealed a good final aesthetic result (Fig. 6A). Although the patient was completely satisfied, she noted that the fluorescence levels of these resins were higher than natural teeth under black light such as in dance clubs with no consequence on dailylife (Fig. 6B).

## 3 Discussion

In adolescents with microdontia, tooth restorations are often carried out after orthodontic treatment to avoid compromising the final outcome [2]. Though this case report, it has been shown that digital workflow merging orthodontic setup and restorative wax-up allowed pre-orthodontic reshaping of anterior teeth. Teeth restorations before starting orthodontic treatment could be beneficial in guiding future teeth movements toward the final occlusion and aesthetics [4,5]. Ideal teeth shape could also restore functional contact points to recreate interdental papillae. However, superimposition of facial and intraoral scans guided by extraoral and intraoral scan body systems could have improved alignment efficiency [24].

In the case currently discussed, digital workflow offered the possibility of reshaping tooth surfaces using bilayering composite resin injection technique, as previously described for two cantilever-type mandibular central incisors restorations [18]. It provided exact thickness measurements of adding materials with more efficient tools compared to conventional waxing process. Composite resin layers have to be less than 2.0 mm thick to avoid composite shrinking during light-curing. As shown in this case, combining flowable composite and injection technique allowed such thin layers of restoration.

The flowable composite used was a flowable-type nano-hybrid composite promoted as materials with improved mechanical and surface properties according to their higher filler content [[11], [12], [13]]. The manufacturers claim that G aenial Universal Flo has higher strength, higher wear resistance and higher gloss retention when compared to currently available leading flowable and conventional composites. A 3-year follow-up study on posterior restorations have shown that their clinical effectiveness was similar to paste-type composite [12]. However, studies or clinical cases have revealed low color stability and surface hardness, requiring to inform patient about regular follow-up for polishing maintenance [19,25]. Moreover, excess fluorescence was identified in this case, which could compromise the aesthetic aspect. It was also reported limitations in polymerization shrinkage of this flowable composite due to highly filler content [26]. This stress reduction could lead to gaps and marginal defects. Recently, Tusi et al. [27] recorded contradictory results showing high polymerization shrinkage of this composite. In the future, more research about these materials is critical.

In this case, flexible clear silicone indexes were made manually using a clear polyvinylsiloxane. To complete digital workflow, 3D printer using direct light processing could have been used to manufacture indexes [21,22]. However, indexes were designed from 3D printed casts. Many studies have analyzed linear measurements on printed models and on stone casts [[28], [29], [30]]. It has been found that 3D printed models were slightly less accurate compared to plaster models [28]. Volumetric changes in conventional method were smaller than those of 3D models [29]. Brown et al. [30] have reported that 3D printers produced models with high accuracy. For these studies, differences found were in the range of clinical acceptance. Prototyped models could be considered accurate enough for composite injection technique. In this case, few occlusal and anatomical adjustments have been made.

To conclude, maxillary lateral incisors and canines were recontoured before an orthodontic treatment to guide future teeth movement. The injection composite technique combined with digital workflow was the restorative approach chosen to obtain a precise anatomy replicating the diagnostic wax-up. This is a simple procedure to create a viable semipermanent reversible restoration from unprepared teeth leading to a good functional and aesthetic outcome. Although the nano-charged composite used is known to present lower color stability and polymerization shrinkage, this technique is a clinical alternative allowing to wait until adulthood, when definitive gold standard prosthodontic solutions are possible.

## 4 Summary

This clinical report described the completely digital workflow used to close diastemas in an adolescent patient before orthodontic treatment. The use of injection technique allowed well-matched tooth-colored restorations with aesthetic benefits, except for fluorescence properties.

## Author contribution statement

All authors listed have significantly contributed to the investigation, development and writing of this article.

## Data availability statement

Data will be made available on request.

## Declaration of competing interest

The authors declare that they have no known competing financial interests or personal relationships that could have appeared to influence the work reported in this paper.
